# Helping people access benefits: Millions of unclaimed federal dollars are available

**DOI:** 10.17269/s41997-025-01064-y

**Published:** 2025-06-17

**Authors:** Noralou P. Roos, Sharon Macdonald, Eileen Boriskewich, Leslie L. Roos, Sally Massey-Wiebe, Colleen J. Metge

**Affiliations:** 1 Department of Community Health Sciences, Manitoba Centre for Health Policy, University of Manitoba, Winnipeg, MB Canada; 2 Community Financial Counselling Services, Winnipeg, MB Canada

**Keywords:** Health, Poverty, Benefits, Interventions, Physicians, Action-oriented, Santé, Pauvreté, Prestations, Interventions, Médecins, Pragmatisme

## Abstract

**Setting:**

The *GetYourBenefits!* Project began as an attempt to convince physicians that it is important to diagnose and treat poverty.

**Intervention:**

The academics worked with community agencies and physician organizations to communicate about the government benefits for which individuals with low incomes and/or disabilities are eligible. The Project Manager and Outreach Officer met with and gave talks to community groups.

The Financial Literacy and Empowerment Program Coordinator, Community Financial Counselling Services (CFCS), who leads Manitoba’s free tax filing clinics, led the development of the Get Your Benefits booklet. The authors decided communicating about the project was important. The project was funded by the Winnipeg Foundation with the collaboration of the Manitoba government and is being continued by CFCS.

**Outcomes:**

This paper describes how information on accessing benefits has been communicated to physicians, health care providers, and those who work in public health. Over 170,000 booklets were distributed. By the final year of the project (2023), over 85 websites had linked to the project website, a major growth over the nine websites linked in the first year of the project. Several updates a year were sent advising on opportunities for accessing benefits, with more than 270 individuals and organizations receiving these in the last year of the project.

**Implications:**

Accessing these benefits has brought and could bring additional millions of unclaimed federal dollars to eligible individuals across Canada. There is still much to be done.

## Résumé

**Lieu:**

Le projet *Demandez vos prestations!* visait au départ à convaincre les médecins de l’importance de diagnostiquer et de traiter la pauvreté.

**Intervention:**

Des universitaires ont travaillé avec des organismes de proximité et des regroupements de médecins à faire connaître les prestations gouvernementales auxquelles ont droit les personnes à faible revenu ou handicapées. La gestionnaire et l’agent de liaison du projet ont rencontré des associations locales et leur ont donné des présentations.

La coordonnatrice du programme de littératie financière et d’autonomisation de Community Financial Counselling Services (CFCS), un organisme qui dirige les cliniques gratuites de production de déclarations de revenus du Manitoba, a piloté l’élaboration de la brochure « *Demandez vos prestations*». Les autrices ont décidé qu’il était important de faire connaître le projet. Financé par la Winnipeg Foundation avec la collaboration du gouvernement provincial, ce projet est aujourd’hui poursuivi par CFCS.

**Résultats:**

Nous décrivons comment les informations sur les moyens de toucher des prestations sont communiquées aux médecins, aux professionnels de santé et aux personnels de santé publique. Plus de 170,000 brochures ont été distribuées. La dernière année du projet (2023), plus de 85 sites Web étaient liés à celui du projet, alors qu’il n’y en avait que neuf la première année. Plusieurs mises à jour annuelles ont été envoyées pour communiquer les possibilités d’obtenir des prestations; la dernière année du projet, plus de 270 personnes et organismes en ont reçues.

**Implications:**

À l’échelle du Canada, la connaissance de l’existence de ces prestations peut permettre aux personnes admissibles de toucher des millions de dollars de fonds fédéraux non réclamés. Il reste encore beaucoup de travail à faire.

## Introduction

What is one of the most needed and radical changes that health care providers can undertake to improve their patients’ health? Social prescribing, which includes connecting patients to benefits for which they are eligible and which can improve their life, is growing nationally and internationally (Calderón-Larrañaga et al., 2021). Physicians and their colleagues must understand the importance of connecting their patients to benefits available from federal and provincial governments that help low-income patients access income they are eligible for and which will help them to afford necessities that support health. We are part of a *GetYourBenefits!* project which developed insights into how to do this.

Our research at the Manitoba Centre for Health Policy repeatedly has found a strong relationship between poverty and health. Those living in the poorest areas of Winnipeg had a life expectancy of 67 years compared with 84 to 85 years for residents of the affluent suburbs.

Most people living in low-income areas do not seem to die because of poor access to medical care. Those residing in the 20% of the urban areas (Winnipeg and Brandon) with the lowest average household incomes are much more likely to be hospitalized than are residents of the higher income areas.

When physician contacts were examined, individuals in the lowest income quintile made up a somewhat higher percent of the highest users than did their counterparts in the highest income quintile (Chateau et al., 2017).

## Treating poverty: Connect patients to benefits they are eligible for

Inspired by this evidence, in Manitoba we developed the *Get Your Benefits!* project, funded by the Winnipeg Foundation, to encourage physicians and health care providers to diagnose poverty (by asking patients “do you ever have trouble making ends meet at the end of the month” (Bloch & Rozmovits, 2021)), and to treat poverty by connecting people to benefits for which they are eligible. The work was inspired by Gary Bloch, a physician, who runs an inner-city medical clinic in Toronto. His opinion editorial in the Globe and Mail said, “this tax season I will also spend time prescribing tax returns” (Bloch, 2013).

By connecting with key groups in other provinces including New Brunswick and British Columbia, we learned what was working and what might make sense to try. New Brunswick Economic and Social Inclusion Corporation was a crown corporation running income tax clinics, accessing social insurance numbers and child benefits. The PLAN Institute (https://planinstitute.ca/) is a Canada-wide organization which helps groups across the country access disability benefits. We built on their experience and advice and focused on several key initiatives to help people access benefits.

## Income tax filing

For those individuals coping with poverty, probably the most important benefit to prescribe is filing taxes. Filing taxes makes an individual potentially eligible to receive all the income withheld when wages were paid. A parent may be able to increase his/her income dramatically by filing taxes and applying for the Canada Child Benefit. The level of income reported as well as the number and age of the children will determine the amount received; for a family with two children, this might be as high as $13,000 per year. Depending on income source and amounts, the parent might also be eligible for other benefits including the Manitoba Child Benefit, Canada Worker Benefits, and provincial tax credits such as the Residential Renter’s Credit. Additional Manitoba Government benefit programs such as the non-Employment and Income Assistance (EIA) Rent Assist program could also provide supplemental monthly income to families and individuals, including seniors, who do not live in subsidized housing or rely on the resources of the EIA program. Depending on annual income, this can often mean hundreds of additional dollars each month. Almost one in five Canadians with low incomes does not file a tax return (Robson & Schwartz, 2020).

The Community Financial Counselling Services (CFCS) which runs the largest Community Volunteer Income Tax Program (CVITP) in Manitoba, in 2022/2023, completed 14,691 tax returns yielding net refunds/credits of $39,497,260 to those living with low incomes; this averaged $2748 per return filed. CFCS developed a Benefits Reference Sheet which enabled volunteers to identify federal and provincial benefits which clients filing taxes were likely eligible for and how to access them. We promoted this service, encouraging physicians to post posters about tax-filing in their office and hosted nursing student interns who distributed GYB resources to community agencies. The GYB Project Manager and Outreach Officer also spent many hours in urban and rural Manitoba communities giving presentations to seniors, urban resource centres, adult education centres, schools, rural health authorities, child and family service centres, etc., on the resources included in the booklet. Several schools and health authorities have staff designated to promote resources to help families understand the benefits they may be eligible for. We had many requests from them for copies of the GYB booklet as they said it was a great resource tool!

Such free tax clinics are offered across the country to Canadians having a modest income and a simple tax situation (www.canada.ca/taxes-help). According to the Canada Revenue Agency (CRA), New Brunswick’s CVITP clinics brought back $55 million in 2022. The CRA has launched a public awareness campaign in an attempt to get $1.4 billion in unclaimed balances back into people’s bank accounts (Robertson, 2022). The 2.4 million working-age adults who did not file may have lost a total of $1.7 billion in cash benefits for the 2016 benefit year — mostly in the form of child benefits (Robson & Schwartz, 2020).

## Claiming disability benefits

Another important federal benefit for patients living with severe and prolonged impairments is the Disability Tax Credit (DTC). Physicians and their disability diagnoses are the key path to individuals’ and supporting family members’ ability to claim this non-refundable tax credit. The 2022 federal tax return allowed claimants to offset $8870 of their taxable income, thereby reducing taxes payable, when a claim for the Disability Tax Credit was included on their tax filing. Claimants with little or no taxable income could transfer the credit to a caregiver to optimize their tax outcomes instead. Dunn and Zwicker (2018) have estimated that less than 40% of Canadians eligible for the DTC have taken it up.

Individuals deemed eligible by the CRA for the Disability Tax Credit also qualify for other key benefits. These include the Registered Disability Savings Plan (RDSP) to which the federal government will contribute $1000/year to a low-income earner’s RDSP for up to 20 years (until the person living with disabilities turns 49). The government will also contribute up to $70,000 additional to a RDSP, matching up to 3:1 the amount which the person living with disabilities or their family contributes, depending on the family’s income. Details can be found at: https://www.canada.ca/en/employment-social-development/programs/disability/savings/how-much.html.

Bloch (2022) has noted that “years of working with people who experience disability, most of whom live in poverty and without secure housing, have convinced me that I must address their social situations directly to improve their health.” Bloch stressed that the first step is to assist patients with the application for the DTC. Once qualified, the next step is to advise patients to open an RDSP.

Even for those who have qualified for the DTC, the country-wide take-up rate of the RDSP has been appallingly low. Approximately one-third of eligible Canadians (up to age 59) have a Registered Disability Savings Plan (RDSP)—36.3% in 2022. The rate ranged from 24% in New Brunswick to 43% in BC and 8.1% in Nunavut and 21% in the Northwest Territories. Details can be found at: https://www.canada.ca/en/employment-social-development/programs/disability-savings/reports/2023-annual.html.

The Canada Revenue Agency has posted links for medical practitioners—providing a digital application which practitioners can use to guide them through completing key parts of the DTC application — Disability Tax Credit (DTC) — Canada.ca. CRA provides another link to help individuals understand how to apply for an additional benefit (different from the RDSP)—the Child Disability Benefit—a tax-free monthly payment to families caring for a child under age 18 with a severe and prolonged impairment in physical or mental functions — Child Disability Benefit — Canada.ca.

## Accessing benefits for higher education

The Canada Learning Bond (CLB) is another, undersubscribed federal benefit. Children born after 2004 may be eligible to receive $500 in a Registered Educational Savings Plan (RESP) from the federal government, plus additional payments of $100 per year up to age 15 (for a maximum of $2000). Parents/guardians do not have to make RESP contributions to receive the CLB. A parent with one to three children earning $50,197 or less is eligible for this benefit. The money can be used for any type of post-secondary education after graduation from high school, including college, university, or such other programs as computer training, apprenticeship programs, hairdressing, massage therapy, and many more that are offered by an educational institute designated by the CRA. Funds can stay in the account until the child is approximately 35 years old.

Remarkably few families and children are taking advantage of the Canada Learning Bond despite the significant federal funding provided by this benefit. As Table 1 illustrates, British Columbia and Quebec are the only provinces where half of the children eligible for the CLB have been receiving it. In Nova Scotia, New Brunswick, Saskatchewan, and Manitoba, one-third or fewer of those eligible received it in 2021.

**Table 1:** Canada Learning Bond take-up rate and payment in 2021

Province or territory	Cumulative number of children in receipt of CLB (1)	Cumulative number of children eligible for CLB (2)	CLB take-up rate (1) ÷ (2)	Cumulative net CLB payment (millions)
Nova Scotia	33,342	100,573	33.2%	$33.5
New Brunswick	26,706	81,423	32.8%	$27.3
Quebec	441,096	889,458	49.6%	$434.3
Ontario	638,800	1,526,517	41.8%	$652.3
Manitoba	65,017	204,315	31.8%	$62.0
Saskatchewan	48,284	169,224	28.5%	$43.2
Alberta	216,145	527,708	41.0%	$193.7
British Columbia	239,213	469,823	50.9%	$228.9
**Canada**	**1,736,080**	**4,074,469**	**42.6%**	**$1708.1**

Source: Employment and Social Development Canada, Annual Statistical Review-Canada Education Savings Program (2021), p. 30

Our Get Your Benefits project met with the school superintendent’s organization in Manitoba and with several other school groups to communicate about the CLB. Promoting the CLB was taken up by the Seven Oaks School division in Winnipeg; they held multiple sign-up events for parents. These sign-up events included services ranging from completing applications for social insurance numbers to setting up bank accounts for their children to ride services to the events for families unable to access transportation. As Seven Oaks described their efforts: “Families with 1–3 children, who have a combined/total annual household income of less than $46,605, or less than $52,583 with 4 children, are eligible to collect this FREE money, for their children born after January 1st, 2004. The grant is also retroactive, which means even if you didn’t collect in a year that you were eligible, you can go back and get that money. Since we began our efforts, we have had a doubling of CLB access in Seven Oaks and have seen over $1 MILLION in FREE money being accessed by families in our division.”

The Get Your Benefits project was part of the Canada Learning Bond Champions Network, which worked on sharing experiences for increasing uptake and automatic enrollment of low-income children—a policy which was recently announced in the federal budget! We worked with a local journalist recently to communicate CLB issues (https://www.winnipegfreepress.com/business/2024/05/18/automatic-for-the-children) with the broader public.

## Making the case for provincial government support

Given the millions in federal benefits that provinces are not receiving because benefits are unclaimed, one strategy might be for academics to work with local medical associations and other health provider organizations to encourage provincial funding of workers recruited and organized by community groups to work in primary care practices. In the United States, the Health Extension program has government funding to do this (Fagnan, 2017).

Funded by the Winnipeg Foundation, and working with an expert who organizes the Free Tax Filing clinics at a local community agency, Community Financial Counselling Services (CFCS), the Get Your Benefits project developed a booklet directing people to both federal and provincial benefits and credits they might be eligible for.

Several key contacts in Manitoba ministries agreed to review and update the booklet annually, to provide French translations, and to fund printing of the booklets. They also arranged to feature the booklet on the Ministry of Health website (https://www.gov.mb.ca/health/primarycare/providers/getyourbenefits.html), where hard copies can be ordered by those working for a government agency. Much more is included on the Get Your Benefits website (https://www.getyourbenefits.ca/). During the first year of the project, about 21,000 booklets were printed and distributed; in subsequent years, over 50,000 a year were distributed. Almost half went to individuals requesting them from the provincial health website. While some think a hard copy is old school, many people living on low incomes do not have easy access to the internet and hard copies are important. As part of working with agencies that serve the homeless, the need for a short pocket size version of a list of benefits and phone numbers was emphasized and produced.

The booklets have been distributed by all five Manitoba Regional Health Authorities (RHAs) to their healthcare professionals. The booklet can be found in medical clinics, various health centres, family resource centres, and hospitals. Nurses in hospitals keep the booklet on the side of their desk as a resource. Community organizations that help individuals with unemployment, homelessness, mental health, and addictions also order booklets. It is a great resource for newcomers; many schools share the booklets with the parents/guardians of children attending schools in their division. The booklet was integrated into the University of Manitoba’s Office of Interprofessional Collaboration and students taught to use these resources.

We did a survey contacting agencies “which” received booklets in 2018 and received 91 responses, on their awareness and use of the GYB booklet. In total, 87% were familiar with the booklet and its use in their agency. The most frequent resource people reported referring their clients to was filing income taxes (60%). Other frequent resources in the booklet “which” people were connected to included employment and income assistance, mental health, and benefits for families with children.

The feedback we have received on the *Get Your Benefits* booklet has been largely positive (Table 2).

**Table 2:** Comments received on the Get Your Benefits booklet

Organization	Email quote
Brandon Neighbourhood Renewal Corporation	*I came across one of your booklets and I was wondering if it would be possible to get a bunch of physical copies to hand out to our clients here at the Brandon Neighborhood Renewal. The list of resources is super helpful!*
Healthy Start	*I am wondering if we could get an order of your Get Your Benefits booklets for us to share with our program participants. We work with low-income families and newcomer families and have always found these booklets very helpful. If we could get 50 books it would be greatly appreciated*
CancerCare Manitoba	*I was wondering if we can order more hard copies of It’s a Fact: Better Income Can Lead to Better Health? It’s a very valuable resource to have in our library*
New Directions	*Hi. I would like to access the booklet Get Your Benefits. Can you share the electronic copy and I can print, or should I order printed copies from you? New Directions supports over 600 people this would apply to*
Fort Garry Women’s Resource Centre	*I was wondering if our Centre could get copies of the Get Your Benefits Booklet for our clients? Any amount would be greatly appreciated. These are a great resource*
Opportunities for Employment	*This is a such a FANTASTIC resource and I am so happy to see that it has been updated! The information and resources contained in the booklet have been tremendously helpful to both our staff and the job seekers we work with. I am not sure how many paper copies one organization can request but I hand out at least 30 a month (have been photocopying them for about a year now)! …Could I get 3 boxes please?*
Southern Health-Santé Sud	*I’m a social worker and I have enjoyed the booklet form of “Get Your Benefits” resource that your team has put together and would greatly appreciate if I could have an order for myself and our clinical team. If it is not too much to ask, could we have a minimum of 25 booklets? Thank you!*
Winnipeg Community Health Centre	*I facilitate a Healthy Baby program and refer to the Get Your Benefits booklet often. We would like to get more physical copies if possible. Many of our participants do not have access to the internet and would appreciate a physical copy to refer to*
Northern Central Development	*I am a Settlement Services Facilitator, and I work with newcomers to Canada. Each year I have approximately 200 newcomers enter my office. I always provide each family with a Get Your Benefits Booklet, and I noticed it is now updated May 2022. If at all possible, would I be able to have some of the updated booklets mailed to me at the address below? They are fantastic for newcomers!*

In 2024, Community Financial Counselling Services has taken on responsibility for continuing publication of the “Get Your Benefits” booklet and managing its online availability on the CFCS website.

## Other resources available

While working with interested groups across the country, we also located key resources such as Prosper’s benefitswayfinder.org, which is an excellent online guide to benefits individuals across Canada might be eligible for. The 2024 federal budget committed $60 million over 5 years to Prosper Canada to expand financial help services for people with low incomes through community partners. Approximately four times a year, we sent out *Get Your Benefit* updates to keep all our contacts informed as to which new resources we had found. By the end of the project, 285 individuals/organizations in Manitoba and across Canada were on our Update list.

## Summary

Academics, physicians, and health care providers working with community agencies created a powerful social prescribing campaign for connecting patients to benefits. Physicians played a prominent and effective role in the anti-smoking movement years ago — and still do. The archives of Physicians for a Smoke-Free Canada (PSFC) capture their activities via Newsletters and their submissions in response to regulatory proposals going back to 1999.

The same commitment to social prescribing is needed. Significant federal and provincial benefits are available to those living on low incomes. Not all benefits are easy to access. There is an important role for health care workers and those doing research on the impact of poverty on health to assist those who are eligible. We need to communicate that major federal funding can be accessed and persuade governments to support agencies and social workers to help patients negotiate benefit applications. Clinics and Access Centres can emphasize the importance of filing taxes and the need for volunteers in the free tax clinics. Regional Health Authorities in Manitoba are working to educate health providers about the importance of assisting their patients to find the resources to ease their financial burdens. They also connect with community organizations to provide up-to-date information on resources available through presentations, webinars, and other platforms. These authorities work with community agencies to assist residents to file their taxes.

## Conclusion

The evidence relating poverty to health is not new. However, the availability and importance of accessing benefits through filing taxes and other applications are not well known. Tax collectors are considered to be “nasty guys” who want to take our dollars. Communicating to low-income Canadians the remarkable benefits of tax filing and accessing disability and educational benefits needs to happen — and to happen now. Academics, physicians, and community organizations can speak out, write editorials, and talk to politicians about the importance of social prescribing. The 50% of life that makes Canadians sick needs to be diagnosed and treated. This is an important challenge that we need to take on.

**Implications for policy and practice**

What are the innovations in this policy or program?

- The authors describe how they have developed their contacts and communicated their research to help individuals access benefits they are eligible for — and often do not know about. Community agencies have helped the academics understand how to do this, and they are trying to help other academics, health care providers, and community groups understand why social prescribing is important to do.

What are the burning research questions for this innovation?

- What is responsible for the differences among provinces in the “take-up” of various federal benefits? Millions of federal dollars are not being accessed. How many individuals do you communicate with and potentially could work with who can promote benefits not being accessed?
- What sort of targeting is most efficient/effective? Can case studies (such as what developed in Seven Oaks) show better ways to locate individuals to apply for and receive the benefits for which they might be eligible?

## Data Availability

Not applicable.
